# Impacto do Desconhecimento do Paciente e Fatores Socioeconômicos na Apresentação do Paciente à Intervenção Coronária Percutânea Primária

**DOI:** 10.36660/abc.20210521

**Published:** 2022-07-07

**Authors:** Mohamed Khalfallah, Amany Allaithy, Dina A. Maria

**Affiliations:** 1 Tanta University Tanta Egito Tanta University, Tanta – Egito

**Keywords:** Fatores Socioeconômicos, Intervenção Coronária Percutânea, Infarto do Miocárdio

## Abstract

**Fundamento:**

O desconhecimento do paciente sobre o infarto agudo do miocárdio, suas complicações e os benefícios da revascularização precoce é um ponto crucial na determinação dos desfechos. Além disso, a relação entre fatores socioeconômicos e apresentação do paciente à intervenção coronária percutânea primária (ICPP) não foi totalmente estudada.

**Objetivos:**

Nosso objetivo foi investigar se o desconhecimento do paciente e outros fatores socioeconômicos impactam na apresentação do paciente à ICPP.

**Métodos:**

O estudo compreendeu 570 pacientes com infarto agudo do miocárdio com supradesnivelamento do segmento ST (IAMCSST) revascularizados por ICPP. Os pacientes foram classificados em dois grupos de acordo com o tempo total de isquemia (tempo desde o início dos sintomas do IAMCSST até a dilatação com balão); grupo I: Pacientes com apresentação precoce (1-12 horas). Grupo II: Pacientes com apresentação tardia (>12-24 horas). Fatores socioeconômicos, desfechos clínicos incluindo mortalidade e eventos cardíacos adversos maiores (ECAM) foram avaliados em cada grupo. O valor de p < 0,05 foi considerado estatisticamente significante.

**Resultados:**

Existem diferentes fatores socioeconômicos que afetam a apresentação do paciente à ICPP. A análise de regressão multivariada identificou os preditores socioeconômicos independentes da seguinte forma: baixa escolaridade - OR 4,357 (IC95% 1,087–17,47, p=0,038), isolamento social - OR 4,390 (IC95% 1,158–16,64, p=0,030) e desconhecimento sobre os benefícios da revascularização precoce - OR 4,396 (IC95% 1,652–11,69, p =0,003). A mortalidade e ECAM foram mais altas no grupo II.

**Conclusão:**

O desconhecimento do paciente e o baixo nível socioeconômico foram associados à apresentação tardia para a ICPP, com desfechos mais adversos.

## Introdução

O infarto agudo do miocárdio (IAM) é uma das principais causas de morbidade e mortalidade em todo o mundo. Entretanto, os avanços na terapia trombolítica e na intervenção coronária percutânea primária (ICPP) têm permitido que a grande maioria dos pacientes sobreviva.^1^ Pacientes com IAM enfrentam várias dificuldades que podem influenciar sua capacidade de manejar sua condição de forma otimizada. Em primeiro lugar, o desconhecimento do paciente sobre a natureza da doença, suas complicações e os benefícios da revascularização precoce. Além disso, fatores socioeconômicos como educação, emprego e moradia podem afetar a saúde de uma pessoa. Da mesma forma, barreiras financeiras podem levar à não-adesão a tratamentos e recomendações médicas essenciais.^2^ A privação social impacta na incidência de doenças cardiovasculares; além disso, a sobrevida é reduzida após o IAM em pacientes provenientes de meios sociais desfavorecidos.^3^ As pessoas privadas de um ou mais desses fatores podem ter dificuldade de acesso aos cuidados de saúde, o que pode influenciar sua saúde e bem-estar geral.

O infarto agudo do miocárdio é uma situação de emergência que requer decisões e intervenções rápidas. A ICPP é um método altamente recomendado para restaurar o fluxo sanguíneo rapidamente em pacientes com IAM, com o objetivo de minimizar a necrose miocárdica e melhorar a sobrevida.^4^ Os desfechos da ICPP não dependem apenas da experiência dos operadores ou da capacidade dos centros de ICP, que representam apenas uma pequena porcentagem dos desfechos da ICPP. Entretanto, há muitos fatores esquecidos que afetam os desfechos relacionados ao desconhecimento do paciente e fatores socioeconômicos que determinam a apresentação precoce ou tardia do paciente após o início dos sintomas do IAM. No presente estudo, nosso objetivo foi investigar o impacto do desconhecimento do paciente sobre a natureza do IAM e os diferentes fatores socioeconômicos que podem afetar a apresentação do paciente à ICPP.

## Métodos

O presente estudo é um estudo de coorte prospectivo, com o objetivo de investigar o impacto de diferentes fatores socioeconômicos na apresentação do paciente à ICPP. O estudo foi realizado em uma amostra de conveniência de pacientes adultos com infarto agudo do miocárdio com supradesnivelamento do segmento ST (IAMCSST), submetidos à revascularização por ICPP em nosso Departamento Cardiovascular, no *Tanta University Hospital*, que é um centro terciário para população de toda a província, com capacidade para emergência e alta taxa de fluxo. O perfil da população local é uma mistura de uma pequena porcentagem de indivíduos com educação superior e a maioria da população do país, que tem baixo nível de escolaridade. Os pacientes foram classificados em dois grupos de acordo com o tempo total de isquemia (tempo desde o início dos sintomas do IAM até a dilatação com balão); grupo I: Pacientes com apresentação precoce (1-12 horas). Grupo II: Pacientes com apresentação tardia (>12-24 horas). O consentimento informado foi obtido de todos os participantes nesta pesquisa. Cada paciente possuía um número de código designado para o seu número de telefone e endereço. O estudo foi aprovado pelo Comitê de Ética Local e foi realizado de acordo com os princípios da Declaração de Helsinki II. O IAMCSST foi definido pelos sintomas característicos de dor torácica típica, bem como por uma elevação do segmento ST de 1 mm nas derivações inferiores, ou elevação do segmento ST de 2 mm nas derivações torácicas anteriores em duas derivações contíguas, ou um novo, ou presumivelmente novo, bloqueio de ramo esquerdo.^5^ Pacientes com IAMCSST que receberam terapia trombolítica ou cirurgia de revascularização miocárdica ou se apresentaram tardiamente após 24 horas e pacientes com IAMSSST foram excluídos do estudo.

Todos os pacientes foram submetidos à anamnese completa, especialmente em relação à presença de diabetes mellitus, dislipidemia, hipertensão e tabagismo atual. O histórico de infarto do miocárdio prévio, acidente vascular cerebral e doenças arteriais periféricas prévios foi avaliado. O início da dor torácica antes da hospitalização foi determinado, sendo calculado o intervalo de tempo entre o início da dor torácica até a dilatação com balão. Foram questionados o histórico de uso de medicamentos e sua adesão, incluindo medicamentos anti-hipertensivos, redutores de colesterol e antiplaquetários. Também foi avaliado o status socioeconômico dos pacientes, incluindo nível de escolaridade, renda, isolamento social, estado civil, situação profissional. O Inventário de Depressão de Beck foi utilizado, que consiste em uma medida autorreferida de 21 perguntas sobre a gravidade dos sintomas depressivos, com o escore variando de 0 a 64, onde o normal varia de 0 a 10 e escores de 11 ou mais indicam depressão clínica potencial.^6^ Além disso, outros fatores que podem afetar os resultados foram avaliados, incluindo se o paciente tinha seguro-saúde, início de dor torácica durante a noite, morar longe de prestadores de cuidados de saúde e, finalmente, conscientização sobre os benefícios da revascularização precoce.

Um exame clínico completo, eletrocardiograma de superfície de doze derivações e ecocardiograma transtorácico foram realizados em todos os pacientes. Investigações laboratoriais de rotina foram realizadas em todos os pacientes, incluindo medidas dos níveis de hemoglobina sérica, glicemia aleatória, creatinina sérica e CK-MB. Na hospitalização, os pacientes receberam quatro comprimidos mastigáveis de 300 mg de ácido acetilsalicílico, 600 mg de clopidogrel ou 180 mg de ticagrelor, além de heparina não fracionada por via intravenosa. A ICPP foi realizada por via transfemoral ou transradial, de acordo com a preferência do operador. Dois intervencionistas experientes avaliaram um conjunto de parâmetros, incluindo o vaso culpado, comprimento da lesão-alvo, grau de fluxo TIMI antes e após a ICPP, carga do trombo (leve, moderada ou alta). O uso do cateter de aspiração e inibidores da glicoproteína IIb/IIIa foram registrados. O escore de fluxo TIMI foi definido pelo grau de fluxo na artéria coronária epicárdica. Os graus TIMI foram avaliados como (grau 0) = ausência completa de fluxo além do ponto de obstrução; (grau 1) = algum material de contraste flui distalmente à obstrução, mas a opacificação arterial completa não é alcançada; (grau 2) = opacificação tardia de todo o artéria e (grau 3) = visualização imediata e completa de toda a artéria.^7^

Os desfechos de interesse neste estudo foram a ocorrência de mortalidade ou eventos cardiovasculares maiores, incluindo parada cardíaca, insuficiência cardíaca e choque cardiogênico, que é definido como hipotensão persistente com pressão arterial sistólica menor que 90 mmHg por pelo menos trinta minutos, com características de hipoperfusão tecidual apesar da administração adequada de fluidos.^8^ A nefropatia induzida por contraste é definida como um aumento relativo (≥25%) ou absoluto (≥0,5 mg/dl) na creatinina sérica dos níveis basais até o período de três dias após a exposição ao meio de contraste.^9^ A ocorrência de acidente vascular cerebral, revascularização repetida e reinfarto, definido como recorrência de sintomas isquêmicos com novas alterações eletrocardiográficas sugestivas de reinfarto, foi avaliada. Sangramento maior (sangramento que exigiu internação prolongada ou queda de hemoglobina de pelo menos 3 g/dL) foi registrada.^10^ O fenômeno de *no-reflow* ocorre se o fluxo TIMI na artéria for ≤ 2, apesar da dilatação bem-sucedida e ausência de dissecção, espasmo ou embolização distal observados angiograficamente após a conclusão do procedimento.^11^

### Análise estatística

A análise estatística foi executada utilizando-se o *software* SPSS 23 (SPSS Inc. Versão 2015, IBM SPSS Statistics for Windows, versão 23, Armonk, NY: IBM Corp.). A normalidade de cada variável foi testada pelo teste de Shapiro-Wilk. Os dados quantitativos foram expressos como média ± desvio padrão. Os dados qualitativos foram expressos como frequência e porcentagem. O teste *t* de Student para amostras independentes foi utilizado para comparar variáveis quantitativas com distribuição normal. O teste qui-quadrado (χ^2^) foi utilizado para estudar a associação entre as variáveis qualitativas. Sempre que qualquer uma das células esperadas fosse menor que cinco, utilizou-se o teste exato de Fisher. A análise de sobrevivência foi realizada utilizando-se a estatística de Kaplan-Meier com teste de *log-rank* para expressar a significância. A análise de regressão logística multivariada foi realizada para detectar os preditores socioeconômicos independentes que afetam a apresentação do paciente à ICPP. O valor de *p* bilateral <0,05 foi considerado estatisticamente significativo.

## Resultados

O presente estudo foi realizado com 570 pacientes com IAMCSST e submetidos à revascularização por ICPP. Os pacientes foram divididos em 2 grupos de acordo com o tempo total de isquemia: grupo I: 280 pacientes (49,1%) com apresentação precoce (1-12 horas). Grupo II: 290 pacientes (50,9%) com apresentação tardia (>12-24 horas). Não houve diferença estatisticamente significante entre os dois grupos em relação à idade, distribuição por gênero, presença de hipertensão, dislipidemia e tabagismo atual. O número de pacientes com fibrilação atrial no grupo II foi significantemente maior do que no grupo I. A fração de ejeção do ventrículo esquerdo foi significantemente maior no grupo I do que no grupo II. Em relação aos resultados laboratoriais, os níveis de CK-MB e creatinina sérica foram significantemente menores no grupo I do que no grupo II, como mostrado na Tabela 1.

**Tabela 1:** Características basais, dados ecocardiográficos e laboratoriais de todos os pacientes de ambos os grupos

	Grupo I (n=280) (1-12 horas)	Grupo II (n=290) (12-24 horas)	Valor de p
Idade, anos	57,16±12,01	56,60±12,06	0,574
Sexo masculino, n (%)	139 (49,6%)	146 (50,3%)	0,867
Tabagismo, n (%)	74 (26,4%)	79 (27,2%)	0,827
Hipertensão, n (%)	94 (33,6%)	91 (31,4%)	0,576
Diabetes mellitus, n (%)	84 (30,0%)	91 (31,4%)	0,721
Dislipidemia, n (%)	97 (34,6%)	106 (36,6%)	0,634
IM anterior, n (%)	22 (7,9%)	27 (9,3%)	0,536
AVC anterior, n (%)	9 (3,2%)	8 (2,8%)	0,749
Doença vascular periférica, n (%)	36 (12,9%)	35 (12,1%)	0,776
Fibrilação atrial, n (%)	24 (8,6%)	41 (14,1%)	0,037*
IMC, (kg/m^2^)	25,26±4,01	25,42±4,36	0,638
Uso de medicação anti-hipertensiva, n (%)	84 (30,0%)	76 (26,2%)	0,314
Uso de medicamentos para baixar o colesterol, n (%)	76 (27,1%)	77 (26,6%)	0,873
Uso de medicação antiplaquetária, n (%)	97 (34,6%)	89 (30,7%)	0,314
PA Sistólica, mmHg	125,3±17,85	124,1±20,9	0,462
PA Diastólica, mmHg	77,50±8,20	76,26±9,50	0,096
FEVE, (%)	47,50±4,65	45,86±6,46	0,001*
Hemoglobina, g/dL	11,56±1,48	11,61±1,46	0,646
Glicemia no sangue aleatória, mg/dL	162,5±43,8	160,6±49,9	0,621
Creatinina sérica, mg/dL	1,036±0,23	1,093±0,24	0,006*
CK-MB, U/L	72,53±33,07	81,98±43,47	0,004*
Volume do agente de contraste, (ml)	184,2±69,9	182,2±65,3	0,728

IM: infarto do miocárdio; IMC: índice de massa corporal; FEVE: fração de ejeção do ventrículo esquerdo; CK-MB: creatina quinase banda miocárdica;

* valor de p significante.

Em relação à situação socioeconômica dos pacientes, seguimento médico, adesão à medicação e conscientização sobre os benefícios da revascularização precoce foram comparados. Houve uma diferença estatisticamente significante entre os dois grupos quanto ao número de pacientes atendidos por médico especialista no ano anterior, sendo maior no grupo I. Além disso, o número de pacientes aderentes ao tratamento médico também foi significantemente maior neste grupo. O número de pacientes que sofreu isolamento social foi maior no grupo II do que no grupo I. O número de pacientes com baixo nível de escolaridade foi significantemente maior no grupo II do que no grupo I. Quanto à conscientização dos pacientes sobre os benefícios da revascularização precoce, o número de pacientes conscientes disso foi significantemente maior no grupo I do que no grupo II. O número de pacientes com início dos sintomas durante a noite foi maior no grupo II e o número de pacientes que moravam longe de prestadores de serviços de saúde também foi maior no grupo II, como mostrado na Tabela 2.

**Tabela 2:** Fatores socioeconômicos de todos os pacientes em ambos os grupos

		Grupo I (n=280) (1-12 horas)	Grupo II (n=290) (12-24 horas)	Valor de p
Consultou um especialista médico no ano anterior, n(%)		193 (68,9%)	113 (39,0%)	0,001*
Adesão ao tratamento médico, n (%)		159 (56,8%)	121 (41,7%)	0,001*
Categoria de renda				
	Renda alta, n (%)	88 (31,4%)	77 (26,6%)	0,199
	Renda baixa, n (%)	192 (68,6%)	213(73,4%)	
Nível de escolaridade				
	Bacharelado ou superior, n (%)	119 (42,5%)	88 (30,3%)	0,003*
	Ensino médio ou inferior, n (%)	161 (57,5%)	202 (69,7%)	
Isolamento social				
	Mora acompanhado, n (%)	248 (88,6%)	228 (78,6%)	0,001*
	Mora sozinho, n (%)	32 (11,4%)	62 (21,4%)	
Inventário de depressão de Beck				
	Normal, n (%)	247 (88,2%)	250 (86,2%)	0,473
	Anormal, n (%)	33 (11,8%)	40 (13,8%)	
Estado civil				
	Casado, n (%)	188 (67,1%)	177 (61,0%)	0,129
	Separado/Divorciado/ Solteiro/	92 (32,9%)	113 (39,0%)	
	Viúva/Viúvo, n (%)			
Situação de emprego				
	Empregado, n (%)	173 (61,8%)	170 (58,6%)	0,718
	Aposentado, n (%)	50 (17,9%)	54 (18,6%)	
	Desempregado, n (%)	57 (20,4%)	66 (22,8%)	
Consciente dos benefícios da revascularização precoce, n (%)		179(63,9%)	103 (35,5%)	0,001*
Início da dor torácica durante a noite, n (%)		112 (40,0%)	148 (51,0%)	0,008*
Plano de Saúde, n (%)		89 (31,8%)	81 (27,9%)	0,315
Mora longe dos prestadores de serviços de saúde, n(%)		33 (11,8%)	52 (17,9%)	0,039*

* Valor de p significante.

Em relação aos resultados angiográficos, a carga do trombo na lesão do vaso culpado foi significantemente maior no grupo II do que no grupo I. Além disso, a necessidade de uso de cateter de aspiração e inibidores da glicoproteína IIb/IIIa também foi maior no grupo II. Não houve diferença estatisticamente significante entre os dois grupos em relação ao fluxo TIMI inicial, ao comprimento da lesão ou ao vaso culpado, embora o fluxo TIMI pós-procedimento tenha apresentado diferença estatisticamente significante, com maior incidência de *no-reflow* no grupo II, como mostrado na Tabela 3.

**Tabela 3:** Resultados angiográficos de todos os pacientes de ambos os grupos

		Grupo I (n=280) (1-12 horas)	Grupo II (n=290) (12-24 horas)	Valor de p
Intervalo desde o início dos sintomas até o PCM, (horas)		7,61±2,71	18,34±3,41	0,001*
Intervalo do PCM até a dilatação com balão, (minutos)		63,98±19,50	64,04±19,45	0,971
Fluxo TIMI inicial				
	0-2	246 (87,9%)	265 (91,4%)	0,168
	3	34 (12,1%)	25 (8,6%)	
Fluxo TIMI pós-procedimento				
	0	2 (0,7%)	7 (2,4%)	0,027*
	1	8 (2,9%)	18 (6,2%)	
	2	13 (4,6%)	22 (7,6%)	
	3	257 (91,8%)	243(83,8%)	
Carga trombótica				
	Baixa	147 (52,5%)	116 (40,0%)	0,010*
	Moderada	85 (30,4%)	106 (36,6%)	
	Alta	48 (17,1%)	68 (23,4%)	
Cateter de aspiração		22 (7,9%)	39 (13,4%)	0,031*
Inibidores da glicoproteína IIb/IIIa		26 (9,3%)	48 (16,6%)	0,010*
Tipo de reperfusão				
	Angioplastia com balão	8 (2,9%)	14 (4,8%)	0,466
	Stenting direto	56 (20,0%)	55 (19,0%)	
	Colocação de stent após pré-dilatação	216 (77,1%)	221 (76,2%)	
Comprimento da lesão, mm		21,39±5,40	20,73±5,25	0,143
Vaso culpado				
ACE, n (%)		6 (2,1%)	7 (2,4%)	0,829
ADA, n (%)		111 (39,6%)	121 (41,7%)	0,613
	CX, n (%)	85 (30,4%)	90 (31,0%)	0,861
Artéria coronária direita, n (%)		78 (27,9%)	72 (24,8%)	0,412

PCM: primeiro contato médico; TIMI: trombólise no infarto do miocárdio; ACE: artéria coronária esquerda; ADA: Artéria descendente anterior; CX: artéria circunflexa;

* valor de p significativo.

**Figura 1:**
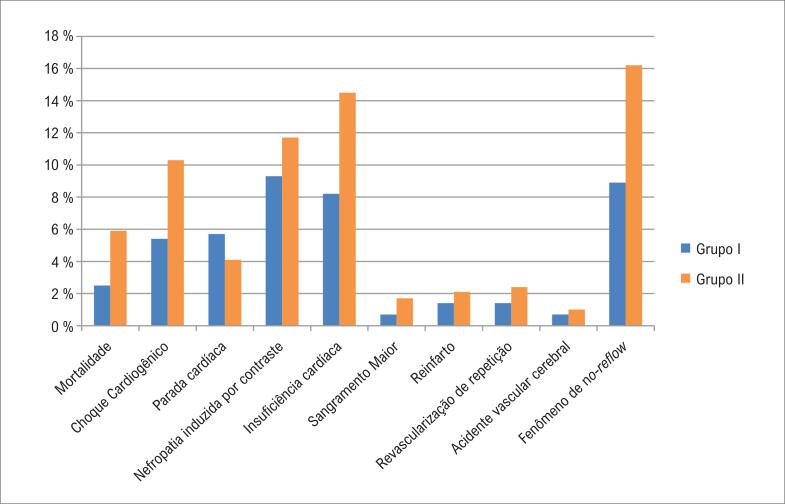
Resultados da intervenção coronária percutânea primária de ambos os grupos.

Em relação aos desfechos, a mortalidade foi significantemente maior no grupo II do que no grupo I. A incidência de choque cardiogênico foi significantemente maior no grupo II do que no grupo I. O número de pacientes com insuficiência cardíaca foi maior no grupo II do que no grupo I. Além disso, a ocorrência do fenômeno de *no-reflow* foi significantemente maior no grupo II do que no grupo I, como mostrado na Tabela 4 e na Figura 1.

**Tabela 4:** Resultados da intervenção coronária percutânea primária

	Grupo I (n=280) (1-12 horas)	Grupo II (n=290) (12-24 horas)	Valor de p
Mortalidade, n (%)	7 (2,5%)	17 (5,9%)	0,046*
Choque cardiogênico, n (%)	15 (5,4%)	30 (10,3%)	0,027*
Parada cardíaca, n (%)	16 (5,7%)	12 (4,1%)	0,384
Nefropatia induzida por contraste, n (%)	26 (9,3%)	34 (11,7%)	0,343
Insuficiência cardíaca, n (%)	23 (8,2%)	42 (14,5%)	0,019*
Sangramento maior, n (%)	2 (0,7%)	5 (1,7%)	0,274
Reinfarto, n (%)	4 (1,4%)	6 (2,1%)	0,560
Revascularização de repetição, n (%)	4 (1,4%)	7 (2,4%)	0,393
Acidente vascular cerebral, n (%)	2 (0,7%)	3 (1,0%)	0,682
Fenômeno de *no-reflow*, n (%)	25 (8,9%)	47 (16,2%)	0,009*

* valor de p significante.

**Figura 2:**
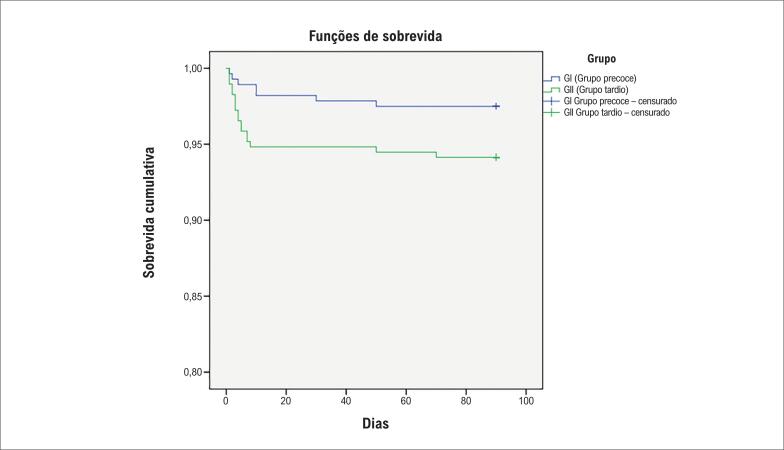
Curva de Kaplan-Meier mostrando sobrevida cumulativa em pacientes dos grupos de apresentação precoce e tardia.

A análise de regressão multivariada foi realizada para identificar os preditores socioeconômicos independentes que afetam a apresentação do paciente à ICPP, como mostrado na Tabela 5, com os seguintes resultados: nível de escolaridade - OR 4,357 (IC95% 1,087–17,47, p=0,038), isolamento social - OR 4,390 (IC95% 1,158–16,64, p=0,030) e conscientização sobre os benefícios da revascularização precoce - OR 4,396 (IC95% 1,652–11,69, p=0,003). A curva de Kaplan Meier foi utilizada, mostrando a sobrevida cumulativa em pacientes de ambos os grupos, como mostrado na Figura 2.

**Tabela 5:** Análise da regressão multivariada para preditores independentes socioeconômicos que afetam a apresentação do paciente à ICPP

	Análise multivariada		Valor de p
	OR	(IC95%)	
Consultou um médico especialista no ano anterior	2,364	0,866–6,450	0,093
Adesão ao tratamento médico	1,237	0,436–3,511	0,689
Nível de escolaridade	4,357	1,087–17,47	0,038*
Isolamento social	4,390	1,158–16,64	0,030*
Consciente do benefício da revascularização precoce	4,396	1,652–11,69	0,003*
Início da dor torácica durante a noite	1,707	0,493–5,909	0,398
Mora longe dos prestadores de serviços de saúde	1,001	0,279–3,598	0,999

* valor de p significante.

## Discussão

O infarto agudo do miocárdio é uma condição de emergência que requer rápida decisão de procurar orientação médica para revascularização precoce e resgate do músculo cardíaco da necrose. Embora a ICPP seja o padrão-ouro para o tratamento de pacientes com IAMCSST, sua principal limitação é o atraso na sua realização. O manejo contemporâneo do IAMCSST é realizado em torno de terapias de reperfusão precoce para reduzir o tamanho do infarto e otimizar os resultados.^12^ A duração da isquemia é um determinante-chave do tamanho do infarto, uma vez que a morte do miócito é diretamente proporcional à duração da oclusão da artéria coronária.^13^ Portanto, o benefício na sobrevida da desobstrução da artéria coronária ocluída está fundamentalmente relacionado ao tempo muito precoce no curso da apresentação do IAMCSST.^14^ Assim, no presente estudo, dividimos os pacientes em dois grupos de acordo com o tempo total de isquemia, considerado a pedra angular para os resultados da ICPP. Embora seja altamente recomendado que o tempo total de isquemia seja encurtado em pacientes com IAMCSST, ele pode variar de acordo com o conhecimento do paciente sobre a doença e outros diferentes fatores socioeconômicos que determinam a apresentação precoce ou tardia aos provedores de serviços de saúde. Embora a política de saúde do estado tenha sido aprimorada nos anos anteriores, com a integração de diferentes modelos de política de saúde, incluindo o programa (*stent for life*) no qual a ICPP está disponível gratuitamente para todo paciente com IAM, independentemente de sua condição socioeconômica, bem como pela integração da Rede de Atendimento de Emergência-CATH-LAB, decidimos investigar os diferentes fatores socioeconômicos e outros fatores relacionados que podem impactar a apresentação do paciente na ICPP.

Neste estudo, os pacientes com apresentação tardia (grupo II) apresentaram um aumento dos níveis da enzima CK-MB, o que indica um aumento da necrose miocárdica devido à longa duração da isquemia, refletindo também na fração de ejeção do ventrículo esquerdo, significantemente menor neste grupo do que no grupo I. Essa diminuição da fração de ejeção pode levar a desfechos adversos, como observado por Ng et al.,^15^ que estudaram 2.648 pacientes com IAMCSST divididos em três grupos de acordo com a função ventricular esquerda: (1) FEVE gravemente comprometida <40%; (2) FEVE moderadamente comprometida 40-50%; e (3) FEVE normal ≥50 %, concluindo que os eventos adversos estão acentuadamente aumentados naqueles com FEVE <40%.

A análise de diferentes fatores socioeconômicos no presente estudo mostrou que o número de pacientes com baixo nível de escolaridade era significantemente maior no grupo II, assim como o número de pacientes que sofriam de isolamento social e moravam sozinhos era maior neste grupo. Além disso, a conscientização dos pacientes sobre os benefícios da revascularização precoce foi significantemente menor nesse grupo, sugerindo as consequências da busca tardia por atendimento médico. Além disso, o número de pacientes do grupo II atendidos por médico especialista no ano anterior e de pacientes aderentes ao tratamento médico foi significantemente menor neste grupo. Em concordância com nossos resultados, Schröder et al.,^16^ observaram que pacientes com maior nível socioeconômico tinham mais conhecimentos sobre o tratamento médico e conseguiam utilizar os prontuários para obter mais informações, enquanto os pacientes com baixo nível socioeconômico parecem não ter conhecimentos sobre o tratamento e têm dificuldade em compreender as informações que lhes são fornecidas. Além disso, o estudo de Roth et al.,^17^ que estudou o papel do ambiente socioeconômico nos desfechos médicos após IAM e incluiu 870 pacientes com IAMCSST submetidos à ICPP no General Hospital of Vienna, demonstrou uma associação entre a distribuição de nível socioeconômico e fatores de risco convencionais, os quais, por sua vez, mostraram um impacto significante na sobrevida de pacientes com IAMCSST. Em concordância com nossos resultados, Jones et al.^18^ estudaram 13.770 pacientes consecutivos submetidos à ICPP em um único centro entre 2005 e 2011, e relataram várias razões possíveis pelas quais o status socioeconômico pode influenciar os resultados da ICPP, observando que o isolamento social foi visto de forma cada vez mais frequente nos indivíduos de baixo nível socioeconômico e tem sido associado a piores desfechos após o IAM. Além disso, Kareem et al.^19^ que investigaram o impacto do nível socioeconômico sobre eventos cardíacos adversos após angioplastia coronariana, concluíram que o baixo nível socioeconômico estava associado a menor adesão à medicação e maior mortalidade após a ICP. Outro fator importante observado no presente estudo é que o número de pacientes que apresentou início da dor torácica durante o horário noturno foi significantemente maior no grupo II. Analisando este grupo em maiores detalhes, verificou-se que se os pacientes estivessem cientes da natureza do IAM, eles teriam chamado a ambulância durante o horário noturno para encaminhamento ao hospital e revascularização precoce por ICPP, ao invés de ficar em casa e esperar para ir ao hospital pela manhã. Isso reflete a relutância dos pacientes em procurar ajuda médica durante a noite devido ao seu desconhecimento.

No presente estudo, os pacientes do grupo II tiveram maior incidência do fenômeno *no-reflow* do que os pacientes do grupo I. Brosh et al.^20^ também relataram diferença significante no tempo porta-balão em pacientes com e sem o fenômeno *no-reflow*. (p=0,000). Além disso, Yip et al.^21^ demonstraram que a taxa de *no-reflow* foi menor em pacientes que foram reperfundidos em menos de 4 horas, e Kirma et al.^11^ verificaram que a reperfusão tardia > 6 horas estava correlacionada com o fenômeno de *no-reflow* (p<0,05), o que está de acordo com nossos resultados. Nos estágios iniciais do IAM, o trombo é rico em trombócitos e é mais fácil de ser tratado com farmacoterapia adjuvante. Além disso, a reperfusão tardia resulta em um trombo intracoronário bem-organizado e, portanto, reduz a probabilidade de atingir o fluxo TIMI 3.^22,23^

Os desfechos após ICPP foram piores no grupo II, já que a mortalidade e os eventos cardíacos adversos maiores foram significantemente maiores neste grupo do que no grupo I. O choque cardiogênico continua sendo a causa mais comum de morte em pacientes hospitalizados com IAMCSST. A incidência de pacientes com choque cardiogênico foi significantemente maior no grupo II (10,3%) em comparação com o grupo I (5,4%). A razão subjacente pode ser a maior necrose celular que ocorre em pacientes com IAMCSST com apresentação mais tardiamente. Assim, níveis maiores de CK-MB foram encontrados no grupo II. O choque cardiogênico tem uma frequência em torno de 7-10%.^24,25^ Está associada a sinais clínicos de hipoperfusão, que incluem diminuição do débito urinário e vasoconstrição periférica. Além disso, a ocorrência de fibrilação atrial foi significantemente maior no grupo II. A fibrilação atrial pode levar à queda do débito cardíaco com maior comprometimento hemodinâmico.^26,27^ Além disso, os níveis de creatinina sérica foram significantemente maiores no grupo II; todos esses fatores aumentam a possibilidade de nefropatia induzida por contraste que, por sua vez, piora os desfechos e aumenta a mortalidade, apesar dos avanços nas estratégias farmacológicas, mecânicas e de reperfusão.^28–31^

## Conclusões

O desconhecimento do paciente sobre a natureza do IAM, suas complicações e os benefícios da revascularização precoce e o baixo nível socioeconômico dos pacientes foram associados à apresentação tardia da ICPP. Os preditores socioeconômicos independentes que afetaram a apresentação da ICPP no presente estudo foram baixo nível de escolaridade, isolamento social e desconhecimento dos benefícios da revascularização precoce.
