# Supplementary material for: Impacto do Desconhecimento do Paciente e Fatores Socioeconômicos na Apresentação do Paciente à Intervenção Coronária Percutânea Primária
Source: Arq Bras Cardiol. 2022 Jul 7;119(1):25–34. [Article in Portuguese] doi: 10.36660/abc.20210521 (PMC9352125; doi:10.36660/abc.20210521)
Supplement: Translation

# Translation: Impact of Patient Unawareness and Socioeconomic Factors on Patient Presentation to Primary Percutaneous Coronary Intervention

**Authors:** Mohamed Khalfallah, Amany Allaithy, Dina A. Maria

**Affiliations:** 1 Tanta Egypt Tanta University, Tanta – Egypt

**Keywords:** Socioeconomic Factors, Percutaneous Coronary Intervention, Myocardial Infarction

## Abstract

**Background:**

Patient unawareness about acute myocardial infarction, its complications and the benefits of early revascularization is a crucial point that determines the outcomes. Moreover, the relationship between socioeconomic factors and patient presentation to primary percutaneous coronary intervention (PPCI) has not been fully studied.

**Objectives:**

Our objective was to investigate whether or not patient unawareness and other socioeconomic factors impact patient presentation to PPCI.

**Methods:**

The study comprised 570 patients with ST-segment elevation myocardial infarction (STEMI) revascularized by PPCI. The patients were classified into two groups according to the total ischemia time (the time from STEMI symptom onset to balloon dilatation); group I: Patients with early presentation (1-12 hours). Group II: Patients with late presentation (>12-24 hours). Socioeconomic factors, clinical outcomes including mortality and major adverse cardiac events (MACE) were evaluated in each group. A p-value < 0.05 was considered statistically significant.

**Results:**

There are different socioeconomic factors affecting patient presentation to PPCI. Multivariate regression analysis identified the independent socioeconomic predictors as following: low educational level - OR 4.357 (CI95% 1.087–17.47, p=0.038), social isolation - OR 4.390 (CI95% 1.158–16.64, p=0.030) and unawareness about the benefits of early revascularization - OR 4.396 (CI95% 1.652–11.69, p=0.003). Mortality and MACE were higher in group II.

**Conclusion:**

Patient unawareness and low socioeconomic status were associated with late presentation to PPCI with more adverse outcomes.

## Introduction

Acute myocardial infarction (AMI) is a leading cause of morbidity and mortality worldwide. However, advances in thrombolytic therapy and primary percutaneous coronary intervention (PPCI) have enabled the vast majority of patients to survive.^1^ Patients with AMI experience various impediments, which may influence their ability to manage their condition optimally. First of all, the patient unawareness about the nature of the disease, its complications and the benefits of early revascularization. Moreover, socioeconomic factors such as education, employment and housing can affect a person's health. Similarly, financial barriers may lead to non-adherence to essential medical therapies and recommendations.^2^ Social deprivation impacts the incidence of cardiovascular diseases; furthermore, survival is reduced following AMI in patients from deprived social backgrounds.^3^ People who are deprived of one or more of these factors may have difficulty accessing health care, and this may influence their overall health status and wellbeing.

Acute myocardial infarction is an emergency situation that requires rapid decisions and intervention. PPCI is a highly recommended method to restore blood flow rapidly for patients with AMI, aiming to minimize myocardial necrosis and improve survival.^4^ The outcomes of PPCI do not depend only on the experience of the operators or the capability of PCI centers, which represents only a small percentage of PPCI outcomes. However, there are many forgotten factors affecting the outcomes related to patient unawareness and socioeconomic factors that determine patient presentation, either early or late, after AMI symptom onset. In the current study, our objective was to investigate the impact of patient unawareness about the nature of AMI and the different socioeconomic factors that may impact patient presentation to PPCI.

## Methods

The current study is a prospective cohort study, aiming to investigate the impact of different socioeconomic factors on patient presentation to PPCI. The study was conducted on a convenience sample of adult patients with ST-segment elevation myocardial infarction (STEMI), submitted to revascularization by PPCI at the Cardiovascular Department of Tanta University Hospital, which is a tertiary center for people from all over the governorate, with emergency capabilities and a high flow rate. The profile of the local population is a mixture of a small percentage of highly educated individuals and the majority of the population countrywide, who has a low educational level. The patients were classified into two groups according to the total ischemia time (the time from AMI symptom onset to balloon dilatation); group I: Patients with early presentation (1-12 hours). Group II: Patients with late presentation (>12-24 hours). Informed consent was obtained from all participants in this research. Every patient had a code number assigned to his telephone number and address. The study was approved by the local ethical committee and was carried out in agreement with the principles of the Declaration of Helsinki II. STEMI was defined by the characteristic symptoms of typical chest pain, as well as by a 1-mm ST-segment elevation in the inferior leads, or 2-mm ST-segment elevation in the anterior chest leads in two contiguous leads, or a new or presumably new left bundle branch block.^5^ Patients with STEMI who received thrombolytic therapy or underwent CABG or presented later than 24 hours and patients with non-STEMI were excluded from the study.

All patients were submitted to full history taking, especially regarding the presence of diabetes mellitus, dyslipidemia, hypertension and current smoking. History of prior myocardial infarction, previous stroke and peripheral arterial diseases was assessed. The onset of chest pain before admission was determined, then the time interval between chest pain onset to balloon dilatation was calculated. History of medication use and compliance with it was questioned, including antihypertensive, cholesterol-lowering and antiplatelet medications. The socioeconomic status of the patients was assessed, including level of schooling, patients’ income, social isolation, marital and employment status. The Beck Depression Inventory was used, which consists in a 21-question self-reported measure for the severity of depressive symptoms with a score ranging from 0 to 64, where normal scores range from 0 to 10 and scores of 11 or higher indicate potential clinical depression.^6^ Furthermore, other factors that may affect the outcomes were assessed, including whether the patient had health insurance, chest pain onset during the night hours, living away from health care providers and, finally, awareness about the benefits of early revascularization.

A full clinical examination, twelve-lead surface ECG and transthoracic echocardiography were performed in all patients. Routine laboratory investigations including serum hemoglobin, random blood glucose, serum creatinine and CK-MB levels were measured in all patients. On admission, patients received four 300 mg chewable acetylsalicylic acid tablets, 600 mg clopidogrel or 180 mg ticagrelor, in addition to intravenous unfractionated heparin. PPCI was performed via the transfemoral or transradial route consistent with operator preference. Two experienced interventionists evaluated a set of parameters including the culprit vessel, target lesion length, TIMI flow grade before and after the PPCI, and thrombus burden (mild, moderate or high). The use of aspiration catheter and glycoprotein IIb/IIIa inhibitors were recorded. TIMI flow score was defined by the degree of flow into the epicardial coronary artery. TIMI grades were assessed as (grade 0) = complete absence of flow beyond the point of obstruction, (grade 1) = some contrast material flows distal to the obstruction, but complete arterial opacification is not achieved, (grade 2) = delayed opacification of the entire artery and (grade 3) = full prompt visualization of the entire artery.^7^

The outcomes of interest in this study were the occurrence of mortality or major cardiovascular events including cardiac arrest, heart failure, and cardiogenic shock, which is defined as persistent hypotension with systolic blood pressure less than 90 mmHg for at least thirty minutes, with characteristics of tissue hypoperfusion despite adequate fluid administration.^8^ Contrast-induced nephropathy is defined as a relative (≥25%) or absolute (≥0.5 mg/dl) increase in serum creatinine from baseline to 3 days after contrast media exposure.^9^ The occurrence of cerebral stroke, repeat revascularization and re-infarction, which is defined as recurrence of ischemic symptoms with new ECG changes suggestive of re-infarction were assessed. Major bleeding (bleeding that required prolonged hospital stay or drop of hemoglobin of at least 3 gm/dL) was recorded.^10^ No-reflow phenomenon occurs if TIMI flow in the artery is ≤ 2, despite the successful dilation and absence of dissection, spasm or distal embolization seen angiographically after completing the procedure.^11^

### Statistical analysis

Statistical analysis was performed using SPSS 23, (SPSS Inc. Released 2015. IBM SPSS statistics for windows, version 23, Armonk, NY; IBM Corp.). The normality of each variable was tested by Shapiro-Wilk test. Quantitative data were expressed as mean ± standard deviation. Qualitative data were expressed as frequency and percentage. Independent-samples Student's t-test was used to compare normally distributed quantitative variables. The Chi-square test (χ^2^) was used to study the association between qualitative variables. Whenever any of the expected cells were less than five, Fisher's exact test was used. Survival analysis was performed using Kaplan-Meier statistics with log-rank test to express the significance. Multivariate logistic regression analysis was performed to detect the independent socioeconomic predictors affecting patient presentation to PPCI. A two-sided p-value <0.05 was considered statistically significant.

## Results

The current study was carried out with 570 patients presenting with STEMI and submitted to PPCI revascularization. Patients were divided into 2 groups according to total ischemia time; group I: 280 Patients (49.1 %) with early presentation (1-12 hours). Group II: 290 Patients (50.9 %) with late presentation (>12-24 hours). There was no statistically significant difference between the two groups regarding age, sex distribution, presence of hypertension, dyslipidemia and current smoking status. The number of patients with atrial fibrillation in group II was significantly higher than ingroup I. Left ventricular ejection fraction was significantly higher in group I than in group II. Regarding the laboratory results, CK-MB and serum creatinine levels were significantly lower in group I than in group II, as shown in Table 1.

**Table 1:** Basal characteristics, echocardiographic data and laboratory data of all patients in both groups

	Group I (n=280) (1-12 hours)	Group II (n=290) (>12-24 hours)	p-value
Age, years	57.16±12.01	56.60±12.06	0.574
Male gender, n (%)	139 (49.6%)	146 (50.3%)	0.867
Smoking, n (%)	74 (26.4%)	79 (27.2%)	0.827
Hypertension, n (%)	94 (33.6%)	91 (31.4%)	0.576
Diabetes mellitus, n (%)	84 (30.0%)	91 (31.4%)	0.721
Dyslipidemia, n (%)	97 (34.6%)	106 (36.6%)	0.634
Prior MI, n (%)	22 (7.9%)	27 (9.3%)	0.536
Previous stroke, n (%)	9 (3.2%)	8 (2.8%)	0.749
Peripheral vascular disease, n (%)	36 (12.9%)	35 (12.1%)	0.776
Atrial fibrillation, n (%)	24 (8.6%)	41 (14.1%)	0.037*
BMI, (kg/m^2^)	25.26±4.01	25.42±4.36	0.638
Anti-hypertensive medication use, n (%)	84 (30.0%)	76 (26.2%)	0.314
Cholesterol lowering medication use, n (%)	76 (27.1%)	77 (26.6%)	0.873
Anti-platelet medication use, n (%)	97 (34.6%)	89 (30.7%)	0.314
Systolic BP, mmHg	125.3±17.85	124.1±20.9	0.462
Diastolic BP, mmHg	77.50±8.20	76.26±9.50	0.096
LVEF, (%)	47.50±4.65	45.86±6.46	0.001*
Hemoglobin, g/dL	11.56±1.48	11.61±1.46	0.646
Random blood glucose, mg/dL	162.5±43.8	160.6±49.9	0.621
Serum creatinine, mg/dL	1.036±0.23	1.093±0.24	0.006*
CK-MB, U/L	72.53±33.07	81.98±43.47	0.004*
Volume of contrast agent,(mL)	184.2±69.9	182.2±65.3	0.728

MI: myocardial infarction; BMI: body mass index; LVEF: left ventricular ejection fraction; CK-MB: Creatine kinase myocardial band;

* significant p-value.

The patients’ socioeconomic status, medical follow up, compliance with medication and awareness about the benefits of early revascularization were compared. There was a statistically significant difference between the two groups regarding the number of patients seen by medical specialist in the previous year, which was higher in group I. Moreover, the number of patients compliant with medical treatment was also significantly higher in this group. The number of patients who suffered from social isolation was higher in group II than in group I. The number of patients with low level of schooling was significantly higher in group II than in group I. Regarding patient awareness about the benefits of early revascularization, the number of patients who was aware was significantly higher in group I than in group II. The number of patients experiencing symptom onset during the night hours was higher in group II, and the number of patients living away from health care providers was also higher in group II, as shown in Table 2.

**Table 2:** Socioeconomic factors of all patients in both groups

		Group I (n=280) (1-12 hours)	Group II (n=290) (>12-24 hours)	p-value
Has seen a medical specialist in the previous year, n (%)		193 (68.9%)	113 (39.0%)	0.001*
Compliance with medical treatment, n (%)		159 (56.8%)	121 (41.7%)	0.001*
Income category				
	High income, n (%)	88 (31.4%)	77 (26.6%)	0.199
	Low income, n (%)	192 (68.6%)	213(73.4%)	
Level of schooling				
	Bachelor's degree or higher, n (%)	119 (42.5%)	88 (30.3%)	0.003*
	High school or less, n (%)	161 (57.5%)	202 (69.7%)	
Social isolation				
	Lives with others, n (%)	248 (88.6%)	228 (78.6%)	0.001*
	Lives alone, n (%)	32 (11.4%)	62 (21.4%)	
Beck Depression Inventory				
	Normal, n (%)	247 (88.2%)	250 (86.2%)	0.473
	Abnormal, n (%)	33 (11.8%)	40 (13.8%)	
Marital Status				
	Married, n (%)	188 (67.1%)	177 (61.0%)	0.129
	Separated/Divorced/Single/	92 (32.9%)	113 (39.0%)	
	Widow/Widower, n (%)			
Employment status				
	Employed, n (%)	173 (61.8%)	170 (58.6%)	0.718
	Retired, n (%)	50 (17.9%)	54 (18.6%)	
	Unemployed, n (%)	57 (20.4%)	66 (22.8%)	
Awareness about the benefits of early revascularization, n (%)		179(63.9%)	103 (35.5%)	0.001*
Onset of chest pain during night hours, n (%)		112 (40.0%)	148 (51.0%)	0.008*
Health insurance, n (%)		89 (31.8%)	81 (27.9%)	0.315
Living away from health care providers, n (%)		33 (11.8%)	52 (17.9%)	0.039*

* significant p value.

Regarding the angiographic results, the lesion thrombus burden in the culprit vessel was significantly higher in group II than in I group. Moreover, the need for aspiration catheter and glycoprotein IIb/IIIa inhibitor use was also higher in group II. There was no statistically significant difference between the two groups regarding initial TIMI flow, the length of the lesion or the culprit vessel, although post-procedural TIMI flow showed a statistically significant difference with a higher incidence of no-reflow in group II, as shown in Table 3.

**Table 3:** Angiographic results of all patients in both groups

		Group I (n=280) (1-12 hours)	Group II (n=290)(>12-24 hours)	p-value
Interval from symptom onset to FMC, (hours)		7.61±2.71	18.34±3.41	0.001*
Interval from FMC to balloon dilation, (minutes)		63.98±19.50	64.04±19.45	0.971
Initial TIMI flow				
	0-2	246 (87.9%)	265 (91.4%)	0.168
	3	34 (12.1%)	25 (8.6%)	
Post-procedural TIMI flow				
	0	2 (0.7%)	7 (2.4%)	0.027*
	1	8 (2.9%)	18 (6.2%)	
	2	13 (4.6%)	22 (7.6%)	
	3	257 (91.8%)	243(83.8%)	
Thrombus burden				
	Low	147 (52.5%)	116 (40.0%)	0.010*
	Moderate	85 (30.4%)	106 (36.6%)	
	High	48 (17.1%)	68 (23.4%)	
Aspiration catheter		22 (7.9%)	39 (13.4%)	0.031*
Glycoprotein IIb/IIIa inhibitors		26 (9.3%)	48 (16.6%)	0.010*
Reperfusion type				
	Balloon angioplasty	8 (2.9%)	14 (4.8%)	0.466
	Direct stenting	56 (20.0%)	55 (19.0%)	
	Stenting after pre-dilation	216 (77.1%)	221 (76.2%)	
Length of the lesion, mm		21.39±5.40	20.73±5.25	0.143
Culprit vessel				
LM coronary artery, n (%)		6 (2.1%)	7 (2.4%)	0.829
LAD coronary artery, n (%)		111 (39.6%)	121 (41.7%)	0.613
	CX coronary artery, n (%)	85 (30.4%)	90 (31.0%)	0.861
Right coronary artery, n (%)		78 (27.9%)	72 (24.8%)	0.412

FMC: first medical contact; TIMI: thrombolysis in myocardial infarction; LM: left main; LAD: left anterior descending; CX: circumflex;

* significant p value.

Concerning the outcomes, mortality was significantly higher in group II than in group I. The incidence of cardiogenic shock was significantly higher in group II than in group I. The number of patients with heart failure was higher in group II than in group I. Moreover, the occurrence of the no-reflow phenomenon was significantly higher in group II than in group I, as shown in Table 4 and Figure 1.

**Table 4:** Outcomes of primary percutaneous coronary intervention

	Group I (n=280) (1-12 hours)	Group II (n=290) (>12-24 hours)	p-value
Mortality, n (%)	7 (2.5%)	17 (5.9%)	0.046*
Cardiogenic shock, n (%)	15 (5.4%)	30 (10.3%)	0.027*
Cardiac arrest, n (%)	16 (5.7%)	12 (4.1%)	0.384
Contrast-induced nephropathy, n (%)	26 (9.3%)	34 (11.7%)	0.343
Heart failure, n (%)	23 (8.2%)	42 (14.5%)	0.019*
Major bleeding, n (%)	2 (0.7%)	5 (1.7%)	0.274
Reinfarction, n (%)	4 (1.4%)	6 (2.1%)	0.560
Repeat revascularization, n (%)	4 (1.4%)	7 (2.4%)	0.393
Cerebral stroke, n (%)	2 (0.7%)	3 (1.0%)	0.682
No-reflow phenomenon, n (%)	25 (8.9%)	47 (16.2%)	0.009*

* significant p value.

**Figure 1:** Outcomes of primary percutaneous coronary intervention in both groups.

Multivariate regression analysis was performed to identify the independent socioeconomic predictors affecting patient presentation to PPCI as depicted in Table 5, with the following results: level of schooling OR 4.357 (CI95% 1.087–17.47, p=0.038), social isolation - OR 4.390 (CI95% 1.158–16.64, p=0.030) and patient awareness about the benefits of early revascularization - OR 4.396 (CI95% 1.652–11.69, p=0.003). The Kaplan Meier curve was performed showing cumulative survival in patients from both groups, as shown in Figure 2.

**Table 5:** Multivariate regression analysis for socioeconomic independent predictors affecting patient presentation to PPCI

	Multivariate analysis		value-p
	OR	(95% CI)	
Has seen a medical specialist in the previous year	2.364	0.866–6.450	0.093
Compliance with medical treatment	1.237	0.436–3.511	0.689
Level of schooling	4.357	1.087–17.47	0.038*
Social isolation	4.390	1.158–16.64	0.030*
Awareness about the benefit of early revascularization	4.396	1.652–11.69	0.003*
Chest pain onset during the night hours	1.707	0.493–5.909	0.398
Living away from health care providers	1.001	0.279–3.598	0.999

* significant p value.

**Figure 2:** Kaplan-Meier curve showing cumulative survival in patients from the early and late presentation groups.

## Discussion

Acute myocardial infarction is an emergency condition that requires rapid decision to seek medical advice for early revascularization and salvage of cardiac muscle from necrosis. Although the PPCI is the gold standard for treating patients with STEMI, its main limitation is the time delay. Contemporary management of STEMI is built around early reperfusion therapies to reduce infarction size and optimize outcomes.^12^ Ischemia duration is a key determinant of infarction size, as myocyte death is directly proportionate to the duration of coronary artery occlusion.^13^ Therefore, the survival benefit from the opening up of the occluded coronary artery is crucially related to the time in the very early course of STEMI presentation.^14^ Therefore, in the current study, we divided the patients into two groups according to the total ischemia time, which is considered the cornerstone for PPCI outcomes. Although it is highly recommended that total ischemia time be shortened in patients with STEMI, it can vary according to the knowledge of the patient about the disease and other different socioeconomic factors that determine the early or late presentation to health care providers. Although the health policy of the state has been enhanced in previous years with the integration of different health policy models, including the program (‘stent for life’) in which PPCI is available freely for all patients with AMI, regardless of their socioeconomic status, as well as by the integration of the Emergency Care Network-CATH-LAB, we decided to investigate the different socioeconomic factors and other related factors that may impact patient presentation to PPCI.

In this study, patients with late presentation (group II) showed an increase in CK-MB enzyme levels, which indicates an increase in myocardial necrosis due to the long duration of ischemia and also reflected on left ventricular ejection fraction, which was significantly lower in this group than in group I. This decrease in ejection fraction can lead to adverse outcomes as reported by Ng et al.,^15^ who studied 2648 patients with STEMI, divided into three groups according to left ventricular function: (1) severely impaired LVEF <40%, (2) moderately impaired LVEF 40–50% and (3) normal LVEF ≥50% and concluded that adverse events are markedly increased in those with LVEF <40%.

The analysis of different socioeconomic factors in the present study showed that the number of patients with low educational level was significantly higher ingroup II, and also the number of patients that suffered from social isolation and lived alone were higher in this group. Moreover, the patients’ awareness about the benefits of early revascularization was significantly lower in this group, implying the consequences of the delayed seeking of medical advice. In addition, the number of patients in group II that was seen by a medical specialist in the previous year and those compliant with medical treatment was significantly lower in this group. In agreement with our results, Schröder et al.,^16^ observed that patients with higher socioeconomic status had greater knowledge about medical treatment and could use medical records to obtain more information, while patients with low socioeconomic status seem to lack knowledge about treatment and have problems in understanding the information provided to them. Moreover, the study by Roth et al.,^17^ who studied the role of the socioeconomic environment on medical outcomes after AMI and included 870 patients with STEMI submitted to PPCI at the General Hospital of Vienna, demonstrated an association between the socioeconomic status distribution and conventional risk factors, which in turn, showed a significant impact on survival for patients with STEMI. In agreement to our results, Jones et al.,^18^ studied 13,770 consecutive patients who underwent PPCI at a single center between 2005 and 2011 and reported several possible reasons why socioeconomic status might influence PPCI outcomes and observed that social isolation was increasingly seen in those of low socioeconomic status and has been associated with poorer outcomes following AMI. Furthermore, Kareem et al.,^19^ who investigated the impact of socioeconomic status on adverse cardiac events after coronary angioplasty concluded that low socioeconomic status, was associated with lower adherence to medication and higher mortality after PCI. Another important factor observed in the present study is that the number of patients who experienced chest pain onset during the night hours was significantly higher in group II. By further analyzing this group, it was found that if patients were aware of the nature of AMI, they would call the ambulance center during the night hours for referral to the hospital and early revascularization by PPCI, rather than staying at home and wait to go to the hospital in the morning. This reflects the patients’ reluctance to seek medical care during the night hours due to their unawareness.

In the current study, patients in group II had a higher incidence of no-reflow phenomenon than patients in group I. Brosh et al.^20^ also reported a significant difference in the door-to-balloon time in patients with and without the no-reflow phenomenon (p=0.000). Moreover, Yip et al.^21^ demonstrated that the rate of no-reflow was lower in patients who were reperfused within less than 4 hours and Kirma et al.^22^ found that delayed reperfusion > 6 hours was correlated with no-reflow (p<0.05), which is in agreement with our results. In the early stages of AMI, the thrombus is rich in thrombocytes and is easier to be treated with adjunctive pharmacotherapy. Furthermore, delayed reperfusion results in a well-organized intracoronary thrombus, thus reducing the likelihood of achieving TIMI 3 flow.^22, 23^

The outcomes after PPCI were worse in group II, as mortality and major adverse cardiac events were significantly higher in this group than in group I. Cardiogenic shock remains the most common cause of death in patients hospitalized with STEMI. The incidence of patients with cardiogenic shock was significantly higher in group II (10.3%) than (5.4%) in group I. The underlying reason may be the fact that more cell necrosis occurs in patients with STEMI that had a later presentation. Thus, the highest CK-MB levels were found in group II. Cardiogenic shock has a frequency of around 7-10%.^24,25^ It is associated with clinical signs of hypoperfusion, which include decreased urine output and peripheral vasoconstriction. Moreover, the occurrence of atrial fibrillation was significantly higher in group II. Atrial fibrillation can lead to a decrease in cardiac output, with more hemodynamic compromise.^26,27^ Furthermore, serum creatinine levels were significantly higher in group II; all of these factors increase the possibility of contrast-induced nephropathy, which in turn worsen the outcomes and increase mortality, despite advances in pharmacological, mechanical and reperfusion strategies.^28–31^

## Conclusions

Patient unawareness about the nature of AMI, its complications and the benefits of early revascularization and the patients’ low socioeconomic status were associated with a late presentation to PPCI. The independent socioeconomic predictors affecting the presentation to PPCI in the current study were low educational level, social isolation and unawareness about the benefits of early revascularization.
